# Imaging characteristics of cardiac metastases in patients with malignant melanoma

**DOI:** 10.1186/s40644-017-0122-8

**Published:** 2017-07-01

**Authors:** Tanja Zitzelsberger, Thomas K. Eigentler, Patrick Krumm, Konstantin Nikolaou, Claus Garbe, Meinrad Gawaz, Bernhard Klumpp

**Affiliations:** 1 Department of Diagnostic and Interventional Radiology, Eberhard Karls University Tuebingen, Hoppe-Seyler-Straße 3, 72076 Tuebingen, Germany; 2 Eberhard-Karls-University Tuebingen, Center for Dermatooncology, Liebermeisterstr. 25, 72076 Tuebingen, Germany; 3 Department of Cardiology, Eberhard Karls University Tuebingen, Tuebingen, Germany

**Keywords:** Malignant melanoma, Metastasis, Heart

## Abstract

**Background:**

Due to prolonged survival and technical advances in CT imaging, cardiac metastases in patients with malignant melanoma are observed more frequently nowadays. The aim of the present study was to assess the anatomic distribution as well as the morphologic and histologic appearance of cardiac metastases from malignant melanoma.

**Methods:**

Twenty five patients with known metastasized melanoma and with incidental finding of cardiac metastases during routine staging CT were retrospectively included in this study. CT images were assessed for the presence, localization and extent of cardiac metastases. Histological results, mutational analysis and tumor markers were reviewed.

**Results:**

Fourteen out of 25 patients presented with singular cardiac mass (56%), whereas ten patients (40%) presented with multifocal and one patient with disseminated cardiac metastases. Twelve patients presented with endocardial (48%), eight with myocardial and two with pericardial metastases. Most frequent site involved in endocardial metastases was the right atrium (67%) followed by the right ventricle (33%). There seems to be a correlation between histological subtype and location of cardiac metastasis. Median survival after diagnosis of cardiac metastases was 8 months, with no significant difference regarding the localization of metastases within the heart.

**Conclusion:**

Cardiac metastases can involve every part of the heart possibly in dependence of histological subtype. The awareness of different types of cardiac metastases and their characteristic appearance on CT images is necessary for further investigations and might contribute to targeted therapy.

## Background

Cardiac metastases are rarely detected in routine staging examinations in patients suffering from malignant melanoma. One reason is that most cardiac metastases remain clinically silent (1). Another reason is that cardiac metastases may evade detection in whole body computed tomography (CT) due to motion artifacts. Yet, cardiac metastases are frequently described in autopsy studies, with an incidence of 2–18% in patients with advanced malignant tumors (2–7). In contrast, the detection of cardiac metastases in vivo is less than 1%, suggesting that despite being rather frequent, most cardiac metastases are only diagnosed post mortem [1–3]. Especially in malignant melanoma cardiac metastases are observed frequently (5, 8, 9). On autopsy, cardiac metastases could be found in as much as 64% of patients with disseminated melanoma (8–10). Due to prolonged survival enabled by advances in systemic therapy, the incidence of cardiac metastases will probably increase. Moreover, technical advances in CT imaging, especially faster image acquisition at increased spatial resolution and improved image contrast may contribute to a more frequent detection of cardiac metastases in these patients. Typical patterns of cardiac dissemination comprise multiple small intramyocardial metastases, but also endocardial metastases could be observed (8, 10). The occurrence of cardiac metastases does not only depend on the ability of melanoma cells to disseminate into the heart but also on the specific histologic and functional environment enabling melanoma cells to settle in the heart (11). Cardiac involvement may arise from hematogeneous or lymphatic spread as well as from direct invasion or venous infiltration (12) and has the potential to affect any portion of the heart [4–10]. Accordingly, clinical symptoms, if present, depend on the anatomic site of the metastases. However, 90% of patients with cardiac involvement are asymptomatic [11, 12]. Clinical symptoms include both dyspnea and peripheral edema due to blood inflow obstruction, arrhythmia, chest pain and pericardial effusion due to tumor infiltration, or thromboembolic events due to thrombus deposition on the surface of endocardial tumor manifestations [13, 14]. These symptoms are unspecific for cardiac metastases and may be difficult to differentiate from other cardiac diseases. Yet, it is of high clinical relevance to identify the source of these symptoms, in order to avert morbidity and mortality arising from cardiac involvement in malignant melanoma.

The aim of the present study was to assess the anatomic distribution as well as the morphologic and histologic appearance of cardiac metastases from malignant melanoma.

## Methods

### Patient group

Patients with known metastasized melanoma and cardiac metastases were retrospectively included in this study. The radiology data base was reviewed to identify melanoma patients diagnosed with cardiac metastases at routine oncological imaging presenting at our hospital. In total 2726 patients were screened covering a period from May 2006 to August 2016. Histological results and patients’ hospital files, including imaging reports and tumor markers (lactate dehydrogenase (LDH) and S100-protein [15]), were reviewed. Patients with cardiac involvement by direct tumor invasion of the heart were excluded as these were regarded not to be primary cardiac metastases.

The study was approved by the institutional review board. Patient consent was waved by the review board as it was a retrospective study.

### CT imaging

In all patients, cardiac metastases from melanoma were incidentally detected during routine CT staging examinations (100%), as none of them suffered from clinical symptoms. All examinations were performed as MSCT (multislice computed tomography) on various Multidetector CT (MDCT) systems (Somatom 4/16/64, Somatom Definition AS and Somatom FLASH (2 × 64 slice), Siemens Healthineers, Forchheim, Germany). Technical parameters were as follows: 250–330 mm field of view with 512 × 512 reconstruction matrix, 120 kV, 100–150 effective mAs and tube rotation time of 0.5/0.3 ms. CT-scans were reconstructed with 3 mm slice thickness coronal and 5 mm slice thickness axial using a soft tissue kernel (B31). Patients received intravenous contrast agent (Ultravist 370, Imeron 400), 2 ml/s, 80–120 ml, adapted to body weight.

### Image analysis

CT images were assessed for the presence, localization and extent of cardiac metastases by two experienced readers (3.5 and 15 year experience in oncological imaging). These were either classified as pericardial metastases, as endocardial metastases, or as myocardial metastases. Metastases along the endocardium with polypoid growth into cardiac cavities were classified as endocardial metastases and these patients were assigned to group 1. Metastases located in the myocardium with infiltrative growth were classified as myocardial metastases and patients were assigned to group 2. Patients with metastases of the pericardium were assigned to group 3. To assess the general tumor burden, the number of involved organs was compared between the patient groups as well as the calculated tumor burden score. As potential site of metastatic spread the following sites were defined: liver, lung, lymph nodes, intestinum, bone, brain, soft tissue, other. The tumor burden score for semiquantitative assessment of tumor burden was defined as follows: one point for one to two metastases per organ, two points for three to five metastases per organ, three points for more than five metastases per organ.

### Statistical analysis

Statistical analyses were performed using SPSS, version 23.0 (IBM Corp., Armonk, NY, USA). Mann Whitney-U-Test was applied for the assessment of gender-specific differences concerning tumor markers. The median of tumor markers and its quartiles were presented using Box Plots. Differences regarding gender and age were assessed using Student’s t-test for independent samples. To compare categorical data of tumor burden for independent subgroups with different sample size a Kruskal Wallis Test was performed. For all tests *p*-values <0.05 were considered to be significant. Median survival after diagnosis of cardiac metastasis was calculated using Kaplan Meier estimators.

## Results

In this retrospective study, 25 patients with known metastasized melanoma were identified with cardiac metastasis. Mean patient age was 58.1 ± 15.6 years (range, 26–89 yeas) and gender distribution was almost equal (male/female: 48%/52%). All patients presented with additional other metastatic sites (extensive disease, Stage IV). There were no statstistical difference between the subgroups (pericardial, myocardial, endocardial and more than one compartment) regarding the involved tumor sites (*p* = 0.141) and general tumor burden (*p* = 0.216). However, results indicate a slightly higher general tumor burden in patients with pericardial metastases as well as in patients with metastases in more than one compartment compared to the other two subgroups.

The histological subtype of melanoma was known in 17/25 patients (68%): superficial spreading melanoma (SSM) in eight patients, nodular melanoma (NM) in four patients, lentigo malignant melanoma (LMM) in two patients, and acral lentiginous melanoma (ALM), mucosal melanoma (MM) as well as melanoma on nevus in one patient, each.

The distribution of primary tumor localization was as follows: head/neck *n* = 3, torso *n* = 9, upper extremity *n* = 1, lower extremity *n* = 4, nasal cavity *n* = 1, and occult primary melanoma *n* = 6. The time between the initial diagnosis of malignant melanoma and the diagnosis of cardiac metastasis was 7.3 ± 8.1 years (range, 0.18–34.7 years). Tumor marker data were available in 20 patients (80%, 8 male). Median level of LDH was higher in male (568 U/l) compared to female patients (248 U/l, *p* = 0,054), without reaching statistical significance. Mean S100 values were also increased in all patients: 0.363 μg/l in male and 0.429 μg/l in female patients, also, with no statistical difference regarding gender (*p* = 0.589). None of the patients suffered from cardiac symptoms; however, in 3 patients, not clinical relevant pericardial effusion was detected by CT.

### CT imaging

Twenty patients received a whole-body examination (80%). 4 patients received thoracic CT (16%) and 1 patient abdominopelvic CT (4%), however, with complete coverage of the heart. CT scan of the thorax was performed in 8 patients (32%) in arterial phase (30 s after contrast agent injection), in 16 (64%) patients in portal-venous phase (90 s after injection) and in 1 patient, only an unenhanced scan of the thorax was performed.

With the exception of pericardial and very large myocardial metastases detection on non-contrast images was impossible. In virtual non contrast images we didn’t find any high density areas attributed to melanin content. At arterial phase as well as portal-venous phase metastases appeared hypodense compared to surrounding myocardium.

For the detection of cardiac metastases portal venous phase seemed to be better suited as cardiac metastases appear as hypodense mass compared to the surrounding myocardium. In arterial phase especially metastases of the right heart are prone to be missed due to dilutational artefacts. In certain cases increased contrast between the ventricular cavity and endocardial metastases in arterial phase might facilitate detection. The presence of metastatic disease was confirmed by follow up examinations. The temporal appearance with new occurrence and increase or decrease in size corresponding to therapy response in other metastatic sites, were regarded to be sufficient in the majority of patients to confirm cardiac metastasis. In uncertain cases additional examinations including echocardiography, PET/CT and MRI were performed to differentiate between metastases and cardiac masses arising from other origin like thrombosis.

#### Anatomic distribution and morphologic appearance

**Fig. 1:**
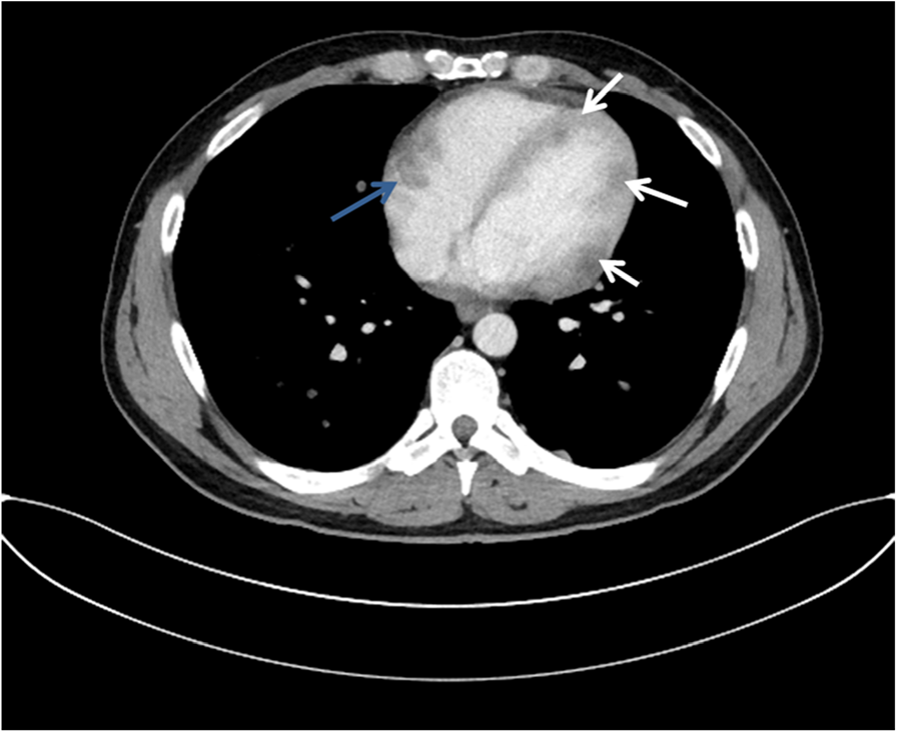
Thirty two year old male patient with multiple myocardial metastases (*white arrows*) as well as endocardial metastasis located in the right atrium (*blue arrow*)

Fourteen patients presented with a singular cardiac mass (56%), whereas 10 patients (40%) presented with multifocal cardiac metastases and 1 patient (4%) with disseminated cardiac metastases. Six patients were found to have metastases in at least two cardiac compartments: there was combination of pericardial and myocardial metastases in one patient, pericardial, myocardial and endocardial in two patients, pericardial and endocardial location in two patients, and myocardial plus endocardial location in one patient. An example of a patient with metastases in two compartments is given in Fig. 1 **.** The histological subtype is known in five out of the six patients with metastases in at least two compartments and was SSM in all of them. Genetic mutational analysis was available in four patients (67%) with two patients showing a mutation in BRAF gene, whereas the other two patients didn’t show any genetic mutation.

**Fig. 2:**
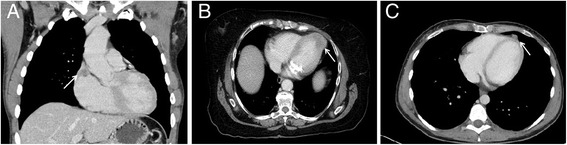
**a** 52-year old male patient with endocardial metastasis located at the junction of vena cava superior – assigned to group 1. **b** 68-year old female patient with myocardial metastasis located at the apex of the left ventricle – assigned to group 2*. The presence of metastatic disease was confirmed by 18F–FDG PET/CT.* **c** 41-year old female patient with pericardial metastasis – assigned to group 3

The distribution analysis of the patients with metastases in only one cardiac compartment identified endocardial metastases in seven patients (36.8%, group 1), myocardial metastases in five patients (26%, group 2) and pericardial metastases in another seven patients (36.8%, group3). Examples of each group are provided in Fig. 2.

The mean lesion size was for endocardial metastases was 31.1 ± 11.3 mm (range 13-50 mm), for myocardial metastases 22.6 ± 8.6 mm (range 13-39 mm) and for pericardial metastases 17.1 ± 8.8 mm (range 8-35 mm).

The most frequent site in group 1 was the right atrium (8 patients, 67%), followed by the right ventricle in 4 patients (33%), the left ventricle in 2 patients (17%), and the left atrium in 1 patient (8%). In group 2, metastases were found in the interventricular septum in 3 patients (37.5%), in the apex of the left ventricle in 4 patients (50%), and in one patient (12.5%) in the lateral left ventricular wall.

Lung metastases were present in 56% of patients with involvement of left ventricular endocardium and myocardium (9/25). In patients (*n* = 4) with involvement of left ventricular endocardium 75% presented with lung metastases. Analysis of general tumor burden shows a slightly higher tumor burden score in patients with pericardial metastasis (mean rank 15.43) and metastases in more than one compartment (mean rank 16.5) compared to the two other subgroups (mean rank 10.5 and 9.36), but with no statistical difference.

For further statistical analysis of the histological subtype, mutational analysis and survival analysis, only patients with metastases in one cardiac compartment (*n* = 19) were included.

Histological subtype was known in twelve patients (63.2%) and missing in seven patients (36.8%); one of group 1, three of group 2 and four of group 3.

In group 1 (*n* = 6), the histological type of the primary tumor was SSM in one patient (16.7%), NM in three patients (50%), ALM in one patient (16.7%), mucosal melanoma in one patient (16.7%).

In group 2 (*n* = 2), the histological type of the primary tumor was LMM and MM on Nevus in one patient (each 50%) each.

In group 3 (*n* = 4), the histological subtype of the primary tumor was SSM in two patients (50%), NM and LMM in one patient (25%) each. Histological subtype in correlation to the localization of metastasis is provided at Table 1.

**Table 1:** Association of histological subtype and localization of cardiac metastasis

	Group 1 endocardial *n* = 6	Group 2 myocardial *n* = 2	Group 3 pericardial *n* = 4
Histological subtype			
SSM (*n* = 3)	33.3%	-	67.7%
NM (*n* = 4)	75%	-	25%
LMM (*n* = 2)	-	50%	50%
ALM (*n* = 1)	100%	-	-
Mucosal (*n* = 1)	100%	-	-
MM on Nevus (*n* = 1)	-	100%	-

Mutational analysis was performed in fourteen patients. Eight patients showed a mutation at BRAF (57%), one at C-Kit (12.5%) and two patients at N-ras (25%). In three patients (37.5%) no mutation was found. The mutation in dependence of the localization of cardiac metastasis is provided at Table 2.

**Table 2:** Association between mutational status and localization of cardiac metastasis

Mutational Analysis			
	BRAF (*n* = 8)	C-Kit (*n* = 1)	N-ras (*n* = 2)
Group 1 – endocardial	37.5%	100%	50%
Group 2 – myocardial	25%	-	50%
Group 3 - pericardial	37.5%	-	-

#### Therapy and clinical outcome

**Fig. 3:**
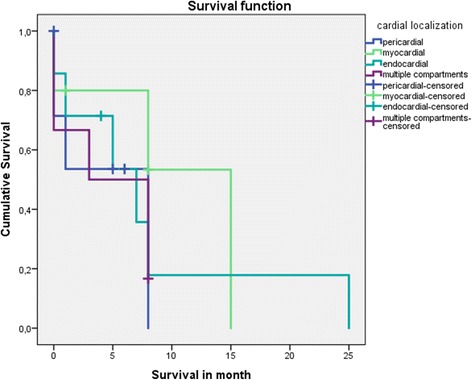
Kaplan-Meier-Estimates of survival for subgroup 1–3

Patients in our study group were treated with various combinations of systemic therapies: check point inhibitors (Vemurafenib), immune related drugs (Ipilimumab, Pembrolizumab, Nivolumab, IL2-Inhibitor), cytotoxic chemotherapy (Carboplatin, Paclitaxel, Temozolomid, Pembrolizumab, Dacarbazin). None of the patients was treated with anticoagulants after the diagnosis of cardiac metastasis. One patient underwent palliative resection of the metastasis, as it was obstructing the vena cava inferior. In all other patients cardiac metastases were not regarded to be the life-limiting factor as most of them had extensive metastatic disease in multiple sites. The overall survival after diagnosis of cardiac metastases was 7.8 +/− 1.9 months (range, 4.1–11.5 months, 28% censored). The mean survival time for patients with pericardial metastases was 4.5 months, with myocardial metastasis 10.1 months, with endocardial metastases 8.2 months and with metastases in multiple compartments 4.5 months. The Kaplan Meier curves for each patient group are provided in Fig. 3. Due to the overlap of Kaplan Meier curves no valid *p*-values can be calculated.

## Discussion

Melanoma is a common neoplasia with increasing incidence [16–18] and a propensity to metastasize to the heart [2, 19]. Cardiac metastases are rare, but 20 to 40 times more common than primary cardiac tumors [20].

While malignant melanoma frequently involves the heart, the diagnosis of cardiac metastases is often late due to the lack of specific clinical signs [1] and limited sensitivity of routine cardiac examinations [21]. Follow up-imaging of malignant melanoma is important for the assessment of therapy response and for the detection of new metastases, potentially indicating the need for additional therapy. Due to its wide availability and favorable cost-effectiveness combined with a high diagnostic accuracy for the detection of metastases, CT is most commonly used for initial staging as well as follow-up examinations in patients with malignant melanoma [22]. While the ante-mortem detection of cardiac metastasis was very rare in the past, enhanced overall survival as well as technical improvements come along with an increased detection rate of cardiac metastases in melanoma patients [23]. Due to the implementation of faster CT scanners, cardiac metastases can be detected using routine whole-body staging protocols enabling exact delineation of cardiac structures even without ECG-gating. Yet, frequent presence of cardiac metastases in autopsy studies and rare detection by CT examinations suggest a significant underestimation of cardiac metastases in malignant melanoma by routine staging techniques.

In our cohort, cardiac metastases were often multifocal and may involve any portion of the heart. According to published data, the pericardium, myocardium and endocardium are involved in descending order [10, 16, 24]. In contrast to published literature pericardial metastases seem to be less frequent in our cohort. Endocardial and myocardial metastases have been detected more often in our study cohort. First of all, technical advances in CT imaging, especially faster image acquisition at increased spatial resolution and improved image contrast may contribute to a more frequent detection of myocardial and endocardial metastases. Moreover the limited patient number restricts the potential to derive data on distribution patterns of cardiac metastases.

Myocardial metastases usually arise from hematogenous spread of singular tumor cells via the coronary arteries and primarily affect the left ventricular myocardium as it represents the biggest portion of total myocardial mass. Myocardial metastases might be more likely to evade detection by CT due to limited soft tissue contrast. Especially small metastases within the cardiac wall may be completely indiscernible. These myocardial metastases may only become apparent by the resulting swelling of the myocardial surface after having reached a size exceeding the regular ventricular wall thickness. In certain patients, myocardial metastases could be identified under therapy due to tumor necrosis presenting as circumscribed hypodense area in contrast-enhanced CT.

In contrast, we found endocardial metastases to be the most frequent, being present in 48% of patients with cardiac metastases. One reason might be a methodical bias, as even small endocardial metastases are well recognizable as polypoid structure protruding into the cardiac cavity, while smaller myocardial metastases might be difficult to detect, as discussed above. Tumor cell deposition within the heart could either occur along the endocardium, preferably in the right atrium and right ventricle, due to metastatic seeding in venous blood draining from parts of the body involved in malignant melanoma. In accordance with published data, this preferably affects the right ventricle and especially the right atrium (6, 17), possibly due to filtration of the blood in the pulmonary capillary vessels reducing the probability of endocardial deposition in the left ventricle [25]. On the other hand, left atrial metastases are more commonly seen in patients with pulmonary metastases whose tumor cells are thought to embolize through the venous circulation of the lung [19]. This assumption is supported by our results, with the right atrium being the most common site involved in 67% of the patients with endocardial metastases, followed by the right ventricle (33%). Nevertheless, consideration should also be given to a potential methodical bias, as motion artifacts are less pronounced in the left and right atrium as well as the right ventricle compared to the left ventricle, which might restrict the detection of metastasis in this cavity.

According to the literature SSM is the most frequent histologic subtype of melanoma composing nearly 59%, followed by NM at 21%, LMM at 11% and ALM at 4% [26]. In contrast, we detected cardiac metastases predominantly in patients with NM, which might be due to the vertical growth behavior and the increased tumor thickness at the time point of diagnosis. Furthermore the assessment of association between histological subtype and localization of cardiac metastasis raised the suspicion that some tumor types preferably affect a special compartment of the heart, e.g., nodular melanoma and endocardial metastases. However, due to the small study population, it is not possible to give a reliable statement. Up to date there are no data on this potential association of histological type and cardiac tumor location.

Cardiac metastases from malignant melanoma usually occur at a state of extensive disease, with a reported five-year survival rate ranging from 9 to 21% in earlier studies [27]. Long-term survival depends on the tumor stage [28], the response to systemic treatment, the surgical resection options and the pre-existing comorbidity of the patient. Due to new therapeutic techniques including tyrosine kinase inhibitors and checkpoint inhibitors, five year survival increased to 37% [29]. In the present study median, survival rate was 8 months after detection of cardiac metastasis. Almost 80% of the patients with cardiac metastases had a mutation of BRAF, NRAS or c-Kit, which might allow for a targeted therapy [30, 31] and potentially extend overall survival. In our study group no significant differences regarding overall tumor burden could be found between the different patient groups. However, results indicate a slightly higher general tumor burden in patients with pericardial metastases as well as metastases in more than one compartment compared to the other two subgroups. This finding may suggest that multicompartment cardiac metastases correlate with more extensive general tumor burden than singular or single compartment metastases.

Still, early diagnosis of cardiac metastases is desirable as in certain locations, palliative surgery might become necessary to avert morbidity and mortality from cardiac failure. In our patient group, one patient necessitated surgical resection of a metastasis as it was located at the right atrium obstructing the inflow of the inferior caval vein (size of metastasis, 33x19mm). Similar strategies for a successful palliative surgery has been described in several case reports [32].

Moreover, anticoagulant therapy should be considered in patients with infiltration of the endocardium, to reduce the potential risk of thromboembolic events. However this could be limited or might be impossible at all due to bleeding of other metastatic sites especially in the gastrointestinal tract, or cerebral metastases with the risk of intracranial hemorrhage. This aspect has to be addressed carefully, as cardiac metastases typically occur only in a far progressed state of metastasized melanoma with a high probability of contraindications for anticoagulant medication, which was the case in almost all of our patients.

Limitations of our study include the retrospective nature as well as the lack of histopathological confirmation of the presence of cardiac metastases. Moreover, the sample size is limited as in vivo diagnosis of cardiac metastases is rare.

## Conclusion

The presence of cardiac metastases in malignant melanoma is presumably underestimated by CT imaging. Cardiac metastases preferably affect patients with aggressive subtypes of malignant melanoma in a progressed state of disease. Dependent on localization and size of cardiac metastases, surgical resection or anticoagulant therapy have to be considered. The awareness of different types of cardiac metastases and their characteristic appearance on CT images might contribute to avert morbidity and mortality in these patients.

## Abbreviations

ALM: Acral lentiginous melanoma
CT: Computed tomography
ECG: Electrocardiography
LDH: Lactate dehydrogenase
LMM: Lentigo malignant melanoma
MDCT: Multidetector computed tomography
MM: Mucosal melanoma
MSCT: Multislice computed tomography
NM: Nodular melanoma
SSM: Superficial spreading melanoma
